# Supplementary material for: A Multi-Omics-Based Exploration of the Predictive Role of MSMB in Prostate Cancer Recurrence: A Study Using Bayesian Inverse Convolution and 10 Machine Learning Combinations
Source: Biomedicines. 2025 Feb 16;13(2):487. doi: 10.3390/biomedicines13020487 (PMC11853722; doi:10.3390/biomedicines13020487)
Supplement: Supplementary file 1 [file biomedicines-13-00487-s001.zip › Figure S1.pdf]

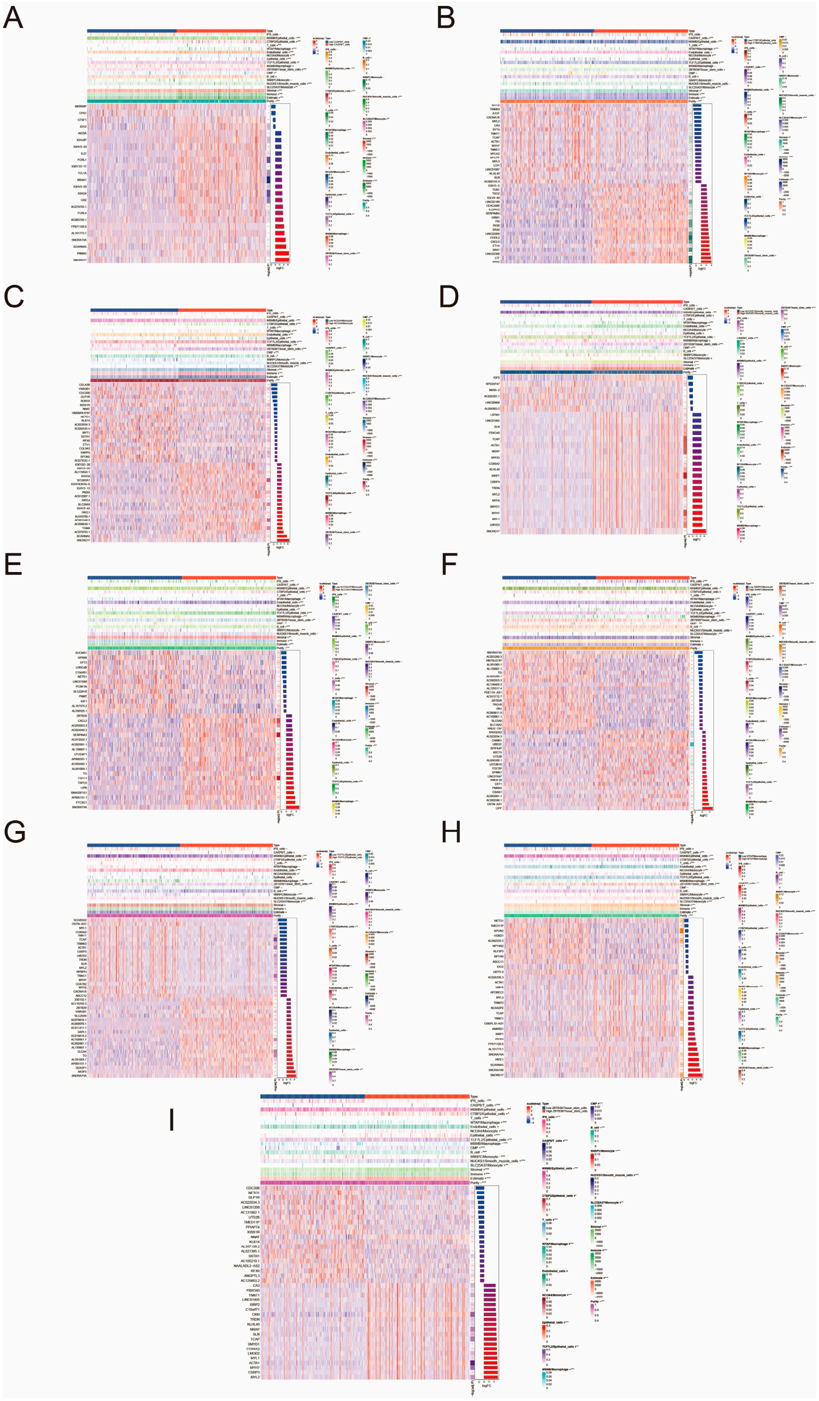

Figure S1. Differential analysis of convolutional cell grouping. (**A**) Heatmap of differential analysis of CASP8/T_cells grouping. (**B**) Heatmap of differential analysis of CTBP2/Epithelial_cells grouping. (**C**) Heatmap of differential analysis of NCOA4/Monocyte grouping. (**D**) Heatmap of difference analysis of NUCKS1/Smooth_stem_cells grouping. (**E**) Heatmap of difference analysis for SLC25A37/Monocyte grouping. (**F**) Heatmap of difference analysis for SNRPC/Monocyte grouping. (**G**) Heatmap of difference analysis of TCF7L2/Epithelial_cells grouping. (**H**) Heatmap of difference analysis for WTAP/Macrophage grouping. (**I**) Heatmap of differential analysis of ZBTB38/Tissue_stem_cells grouping.
