# Supplementary material for: A Multi-Omics-Based Exploration of the Predictive Role of MSMB in Prostate Cancer Recurrence: A Study Using Bayesian Inverse Convolution and 10 Machine Learning Combinations
Source: Biomedicines. 2025 Feb 16;13(2):487. doi: 10.3390/biomedicines13020487 (PMC11853722; doi:10.3390/biomedicines13020487)
Supplement: Supplementary file 1 [file Figure S2.pdf › Figure S2.pdf]

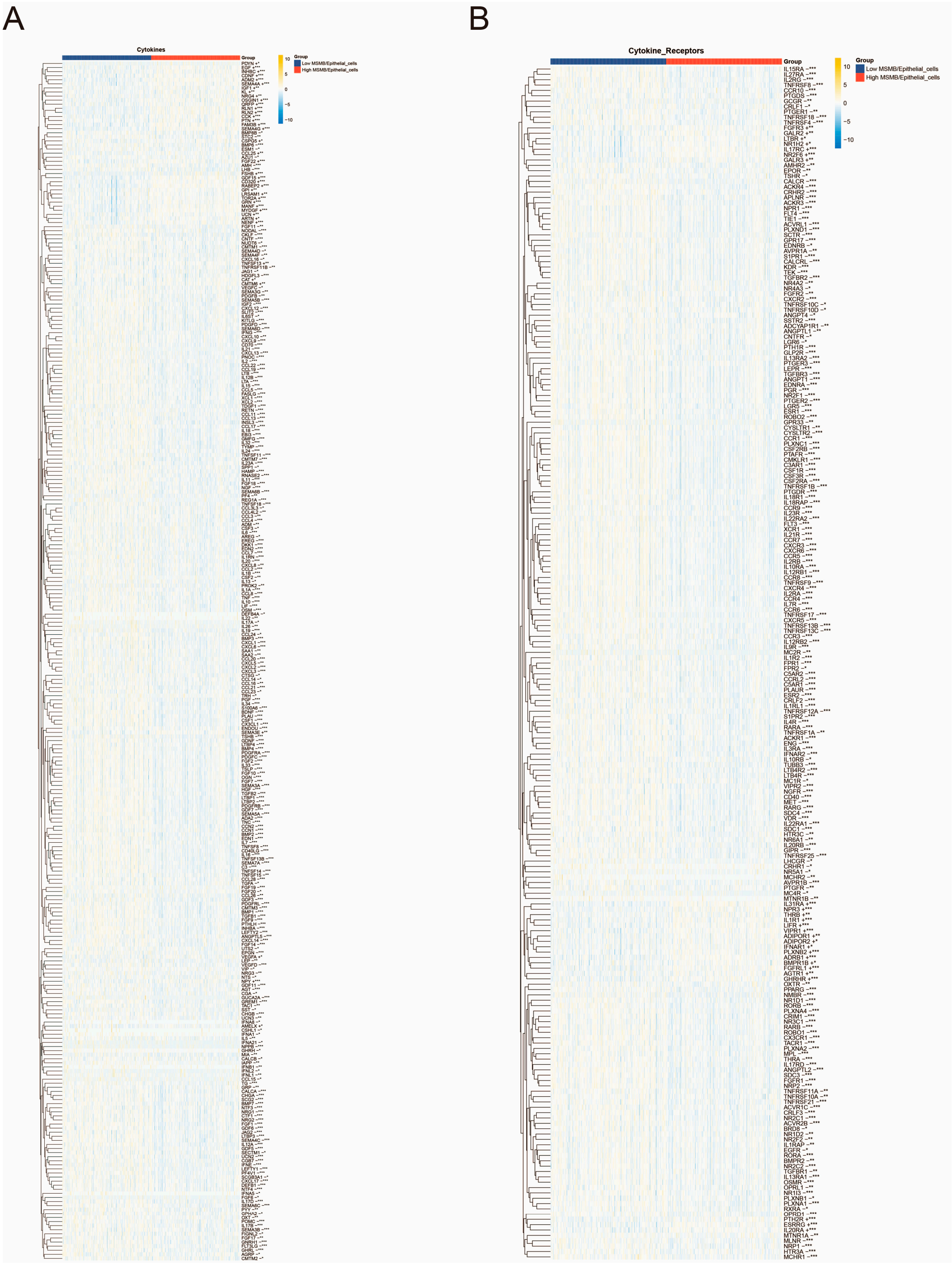

Figure S2. Analysis of cytokines and their receptors in MSMB/Epithelial_cells. (**A**) Cytokine gene set analysis of MSMB/Epithelial_cells. (**B**) Gene set analysis of cytokine receptors of MSMB/Epithelial_cells.
