# Supplementary material for: A Multi-Omics-Based Exploration of the Predictive Role of MSMB in Prostate Cancer Recurrence: A Study Using Bayesian Inverse Convolution and 10 Machine Learning Combinations
Source: Biomedicines. 2025 Feb 16;13(2):487. doi: 10.3390/biomedicines13020487 (PMC11853722; doi:10.3390/biomedicines13020487)
Supplement: Supplementary file 1 [file Figure S3.pdf › Figure S3.pdf]

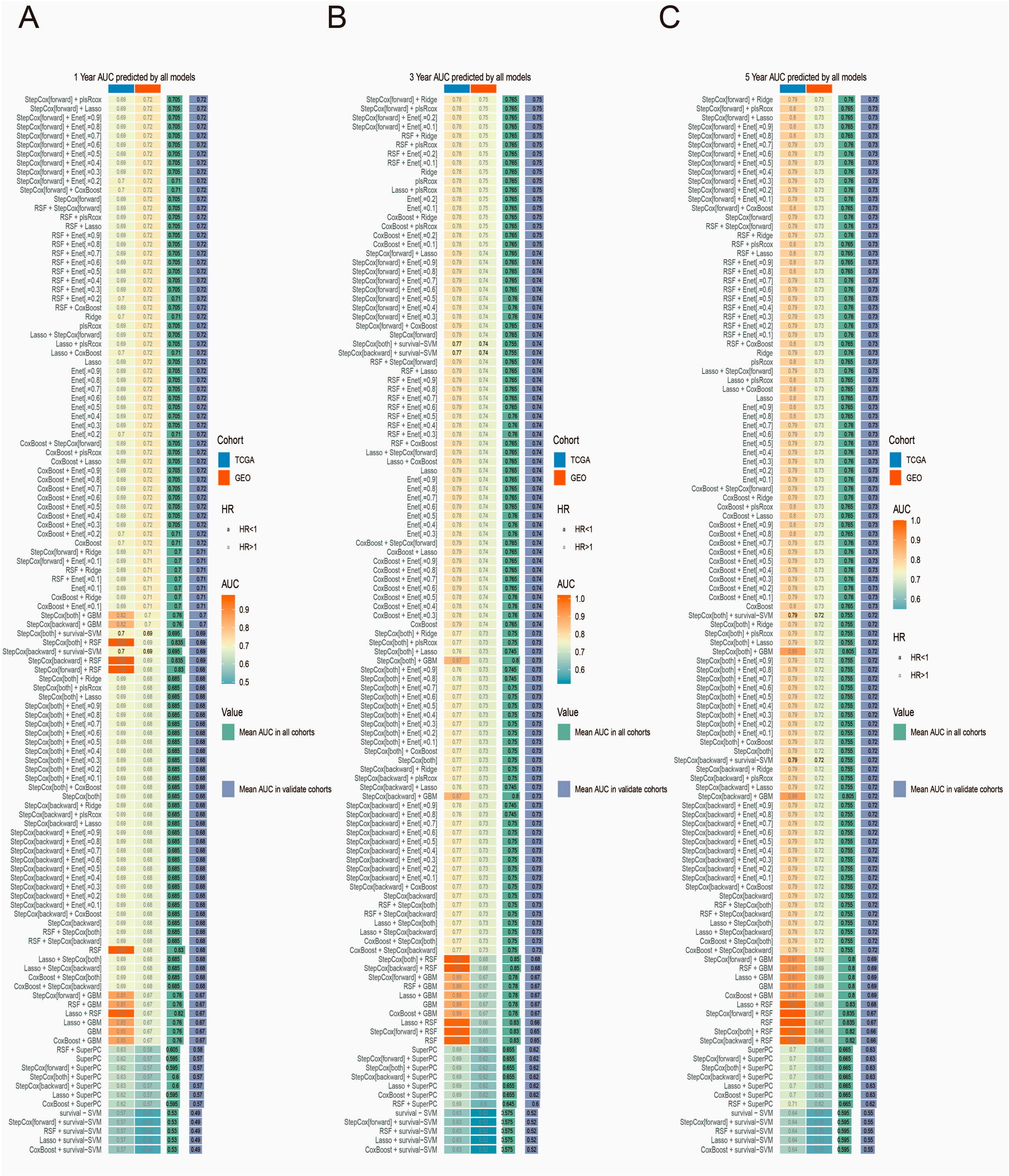

Figure S3. Predicted AUC values for 1, 3, and 5 years based on 10 machine learning. (**A**) AUC values for 1-year survival based on 10 machine learning in the training and validation sets. (**B**) AUC values for 3-year survival based on 10 machine learning in the training set and validation set. (**C**) AUC values for 5-year survival based on 10 machine learning in the training set and validation set.
